# Enhanced Collagen Deposition in the Duodenum of Patients with Hyaline Fibromatosis Syndrome and Protein Losing Enteropathy

**DOI:** 10.3390/ijms21218200

**Published:** 2020-11-02

**Authors:** Jorik M. van Rijn, Lael Werner, Yusuf Aydemir, Joey M.A. Spronck, Ben Pode-Shakked, Marliek van Hoesel, Elee Shimshoni, Sylvie Polak-Charcon, Liron Talmi, Makbule Eren, Batia Weiss, Roderick H.J. Houwen, Iris Barshack, Raz Somech, Edward E.S. Nieuwenhuis, Irit Sagi, Annick Raas-Rothschild, Sabine Middendorp, Dror S. Shouval

**Affiliations:** 1 Division of Pediatrics, Department of Pediatric Gastroenterology, Wilhelmina Children’s Hospital, University Medical Center Utrecht (UMCU), Utrecht University (UU), 3584 CT Utrecht, The Netherlands; jorik.vanrijn@gmail.com (J.M.v.R.); j.m.a.spronck@students.uu.nl (J.M.A.S.); m.vanhoesel-2@umcutrecht.nl (M.v.H.); r.houwen@umcutrecht.nl (R.H.J.H.); e.e.s.nieuwenhuis@umcutrecht.nl (E.E.S.N.); 2 Regenerative Medicine Center, UMCU, UU, 3584 CT Utrecht, The Netherlands; 3 Pediatric Gastroenterology Unit, Edmond and Lily Safra Children’s Hospital, Sheba Medical Center, Ramat Gan 5262100, Israel; wernerlael@gmail.com (L.W.); Batya.Vais@sheba.health.gov.il (B.W.); 4 Sackler Faculty of Medicine, Tel-Aviv University, Tel-Aviv 6997801, Israel; ben_pode@hotmail.com (B.P.-S.); sylvie.polak@gmail.com (S.P.-C.); Liron.Talmi@sheba.health.gov.il (L.T.); iris.barshack@sheba.health.gov.il (I.B.); raz.somech@sheba.health.gov.il (R.S.); araro2013@gmail.com (A.R.-R.); 5 Department of Pediatrics, Division of Pediatric Gastroenterology and Hepatology, Eskisehir Osmangazi University Faculty of Medicine, Eskisehir 26040, Turkey; dryusufaydemir@yahoo.com (Y.A.); makbule99@yahoo.com (M.E.); 6 The Institute for Rare Diseases, Edmond and Lily Safra Children’s Hospital, Sheba Medical Center, Ramat Gan 5262100, Israel; 7 Talpiot Medical Leadership Program, Sheba Medical Center, Ramat Gan 5262100, Israel; 8 Department of Biological Regulation, Weizmann Institute of Science, Rehovot 7610001, Israel; elee.shimshoni@wyss.harvard.edu (E.S.); irit.sagi@weizmann.ac.il (I.S.); 9 Institute of Pathology, Sheba Medical Center, Ramat Gan 5262100, Israel; 10 Pediatric Department A, Edmond and Lily Safra Children’s Hospital, Sheba Medical Center, Ramat Gan 5262100, Israel; 11 Immunology Service, Edmond and Lily Safra Children’s Hospital, Sheba Medical Center, Ramat Gan 5262100, Israel; 12 Jeffrey Modell Foundation Center, Edmond and Lily Safra Children’s Hospital, Sheba Medical Center, Ramat Gan 5262100, Israel

**Keywords:** protein losing enteropathy, intestinal lymphangiectasia, organoids, extracellular matrix, ANTXR2, CMG2

## Abstract

Hyaline fibromatosis syndrome (HFS), resulting from *ANTXR2* mutations, is an ultra-rare disease that causes intestinal lymphangiectasia and protein-losing enteropathy (PLE). The mechanisms leading to the gastrointestinal phenotype in these patients are not well defined. We present two patients with congenital diarrhea, severe PLE and unique clinical features resulting from deleterious *ANTXR2* mutations. Intestinal organoids were generated from one of the patients, along with CRISPR-Cas9 *ANTXR2* knockout, and compared with organoids from two healthy controls. The ANTXR2-deficient organoids displayed normal growth and polarity, compared to controls. Using an anthrax-toxin assay we showed that the c.155C>T mutation causes loss-of-function of ANTXR2 protein. An intrinsic defect of monolayer formation in patient-derived or *ANTXR2^KO^* organoids was not apparent, suggesting normal epithelial function. However, electron microscopy and second harmonic generation imaging showed abnormal collagen deposition in duodenal samples of these patients. Specifically, collagen VI, which is known to bind ANTXR2, was highly expressed in the duodenum of these patients. In conclusion, despite resistance to anthrax-toxin, epithelial cell function, and specifically monolayer formation, is intact in patients with HFS. Nevertheless, loss of ANTXR2-mediated signaling leads to collagen VI accumulation in the duodenum and abnormal extracellular matrix composition, which likely plays a role in development of PLE.

## 1. Introduction

Hyaline fibromatosis syndrome (HFS) is a rare autosomal recessive disorder, called after the deposition of various collagen fibers in the skin and internal organs [1,2,3]. In earlier literature HFS was commonly subdivided into either the milder form, juvenile hyaline fibromatosis, or the more severe and life-limiting form, infantile systemic hyalinosis (ISH), depending on the course of disease. When it was found that both disease phenotypes are caused by mutations in the anthrax toxin receptor 2 (*ANTXR2*) gene [4,5], a new grading system was proposed based on the extent of symptoms [6,7]. Grade 1 (mild) involves skin and/or gingival tissue; grade 2 (moderate) involves joints and/or bones; grade 3 (severe) involves internal organs; grade 4 (life-limiting) presents similar symptoms to grade 3 but significantly limits life expectancy. In most cases, disease onset occurs within the first year of life, characterized by joint stiffness or contracture. Persistent diarrhea is common, affecting 45 of 84 patients (53.6%) in one study that included subjects with different degrees of HFS [8], and all patients in a different study including patients with clinical presentation before 6 months of age and ISH classification at that time [5]. The disease in the severe forms of HFS is fatal within the first two years of life because of intestinal or pulmonary complications [8].

The *ANTXR2* gene, also known as capillary morphogenesis protein 2 (*CMG2*), encodes a single-pass transmembrane receptor which binds its ligand through a von Willebrand A (vWA) domain [1,9]. As the name suggests, the function of this receptor has been well documented as a mode of entry for toxins from the bacteria *Bacillus anthracis*. The physiological role of this receptor, however, remains elusive. ANTXR2 was identified to be upregulated during capillary formation by endothelial cells, where it was found to bind laminin, collagen IV and collagen VI [1,9], suggesting a potential role in basement membrane assembly and extracellular matrix (ECM) formation [9]. This hypothesis is supported by the observation that HFS patient-derived fibroblasts lose their ability to attach to a laminin matrix [5], and more recently that ANTXR2 binds to collagen VI and regulates its degradation [1].

In the severe forms of HFS, protein losing enteropathy (PLE) has been reported, and several patients were noted to suffer from intestinal lymphangiectasia [8,10,11,12,13]. Diverse mechanisms can lead to development of PLE, including aberrant lymphatic formation, mucosal damage or loss of barrier function of the intestinal epithelium [14]. It is, however, unknown if mutations in *ANTXR2* lead to a primary defect of the intestinal epithelium as well, and thus contribute to the clinical phenotype in a multifactorial fashion.

Here we present two patients with typical symptoms of grade 4 HFS. Using intestinal organoids derived from one patient, we studied the primary effects of a novel c.155C>T (p.S52F) missense mutation on the intestinal epithelium. We show that this *ANTXR2* mutation leads to a loss of binding capacity of the vWA domain, as evaluated by an anthrax toxin-dependent cytotoxicity assay. Furthermore, we demonstrate that the barrier function of organoid monolayers is unaffected by loss of functional ANTXR2. Finally, we show abnormal ECM architecture with significant collagen VI accumulation in the duodenum of patients with grade 4 HFS, suggesting that abnormal intestinal ECM deposition is characteristic of this rare disorder, and likely has a role in mediating the clinical phenotype.

## 2. Results

### 2.1. Clinical Phenotype

Patient 1 was a four-months-old male who was transferred to Sheba Medical Center for evaluation of failure to thrive, multiple fractures, intermittent diarrhea and dysmorphic features. The patient was born to an Arab-Muslim couple who were double first-degree cousins (grandfathers were brothers and grandmothers were sisters). Pregnancy was noted for poor growth during the third trimester. He was born by a cesarean section at term weighing 1.7 kg, small for gestational age, and was admitted for two weeks due to fractures of the clavicle and arm, although labor was uneventful. At the age of three months he was readmitted for pneumonia and was found to have multiple fractures and hypoalbuminemia. 

**Figure 1:**
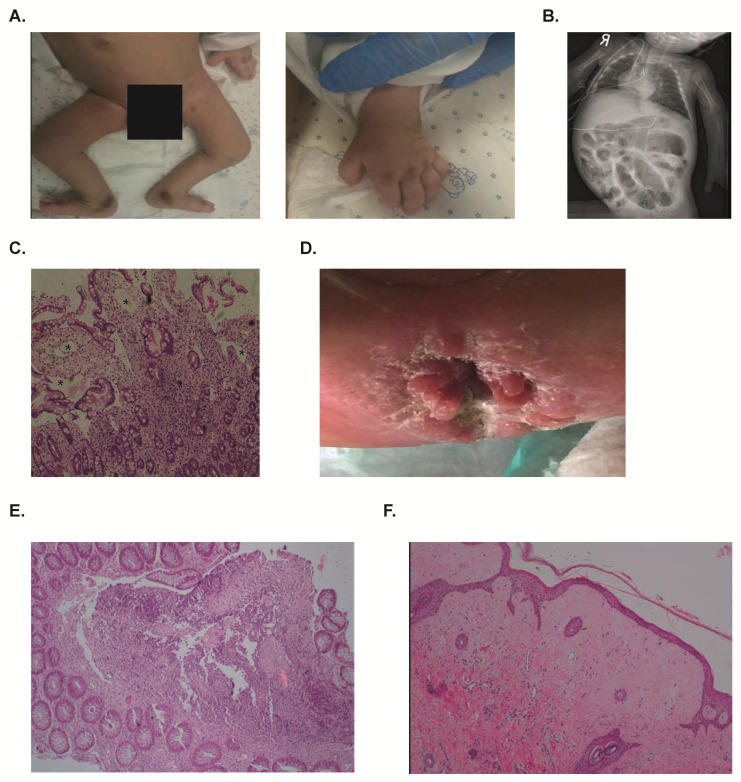
Clinical presentation of patients with ANTXR2 deficiency. (**A**) Hyperpigmentation regions over median malleolus and over the knuckles in both hands of Patient 1. (**B**) Abdominal X-ray of Patient 1 showing marked bowel distention. (**C**) H & E stain of duodenal biopsy of Patient 1 showing distortion of architecture and dilation of lymphatics compatible with lymphangicetasia, marked by asterix. (**D**) Perianal thickened nodules observed in Patient 2. (**E**) H&E stain of colonic biopsy from Patient 2 showing abnormal architecture and marked hyalinosis. (**F**) H&E stain of skin biopsy from Patient 2 demonstrating pink homogeneous acellular material throughout the dermis, suggestive of hyaline.

On physical exam in our institution at the age of 4 months, the patient was cachetic and edematous. Weight was 3.9 kg (−4.6 Z score) and head circumference 32.5 cm (−6.3 Z score). Patient appeared dysmorphic with epicanthal folds, hyperpigmentation over the metacarpophalangeal joints in both hands and over both medial malleoli (Figure 1A) and lower limbs contractures. The patient was extremely irritable and was sensitive to touch. Blood tests showed a normal complete blood count, low albumin levels (2.0 g/dL, normal range 3.5–5.0 g/dL) and normal electrolytes and lipid panel. Urine analysis ruled out proteinuria, while stool alpha-1-anti-trypsin was elevated (90 mg/dL; normal <20mg/dL), suggesting a diagnosis of PLE. A skeletal X-ray demonstrated multiple fractures originating at different ages and osteopenia, while an abdominal X-ray showed markedly distended bowels (Figure 1B). An esophagogastroduodenoscopy (EGD) showed thickened duodenal folds with small white papules, and haematoxylin and eosin (H & E) stains demonstrated blunting of villi and edema of the lamina propria, suggestive of intestinal lymphangiectasia (Figure 1C). D2-40 stain for lymphatic vessels confirmed the presence of intestinal lymphangiectasia in this patient (Supplementary Figure S1). A skin biopsy from this patient demonstrated dermal fibrosis, increased number of fibroblasts and hyalinization of the collagen. 

During his admission, different elemental formulas were provided by naso-gastric tube, including Monogen that has a high content of medium chain triglycerides. However, the patient continued to suffer from diarrhea and did not tolerate these feeds, even when given continuously, and was dependent on total parenteral nutrition (TPN). At the age of nine months he developed septic shock and passed away. 

Patient 2 was a six-months-old male, born to Muslim parents who were first degree cousins, that was evaluated for chronic diarrhea and hypoalbuminemia since infancy. Pregnancy was uneventful and birth weight was 2.8 kg. In the first few weeks after birth he was noted to have deformity of extremities, recurrent episodes of lower respiratory tract infections and diarrhea. On physical examination, the skin was thickened and showed hyperpigmentation over the pressure areas. There was edema at pretibial regions, severe contractures and gingival hypertrophy. In addition, perianal nodules were noted (Figure 1D). Blood tests showed an albumin level of 1.7 g/dL. An EGD revealed diffusely elongated, circumferential and polypoid mucosa covered with whitish enlarged villi at the second portion of duodenum, suggesting intestinal lymphangiectasia, which was confirmed in an H&E stain. Ileocolonoscopy showed mucosal nodularity at the terminal ileum and colon. Histopathological evaluation of colonic tissue samples revealed chronic inflammatory cells and perivascular hyalinosis (Figure 1E). Skin biopsy demonstrated pink homogeneous acellular material throughout the dermis, suggestive of hyaline (Figure 1F). The patient was unable to tolerate different feeds, and eventually passed away due to multi-organ failure at the age of 20 months.

### 2.2. Genetic Studies

**Figure 2:**
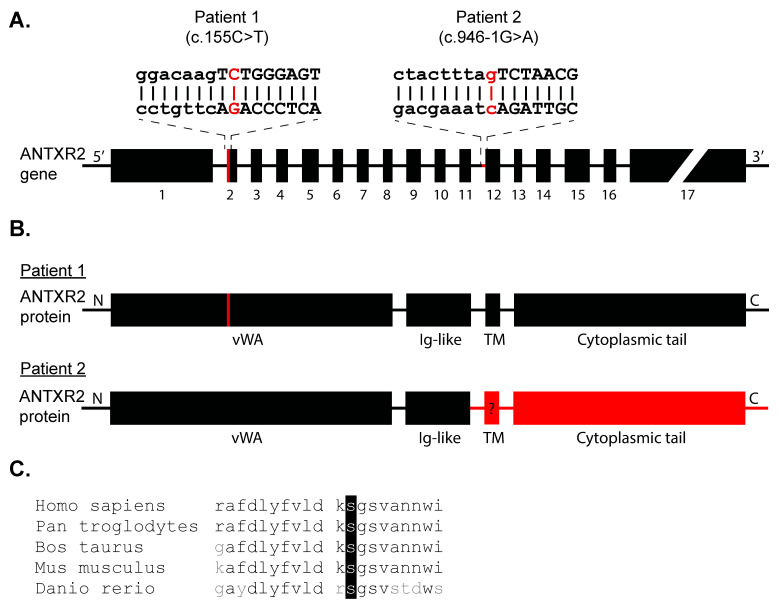
Characteristics of the *ANTXR2* mutations. Schematic of (**A**) the genetic localization of the mutations (indicated in red) and (**B**) the effect of the mutation in the ANTXR2 protein of Patient 1 (c.155C>T, red bar) and Patient 2 (c.946-1G>A). The splice-site mutation of Patient 2 likely leads to loss of protein (deletion indicated in red). (**C**) The mutated serine residue of Patient 1 is highly conserved across among the amino acid sequences of various vertebrates, suggesting critical functional relevance.

Whole exome sequencing of Patient 1 demonstrated a homozygous NM_058172.5 c.155C>T (p.S52F) missense mutation in the metal ion-dependent adhesion site (MIDAS) motif of *ANTXR2′*s vWA domain, a highly conserved amino acid sequence (Figure 2A–C). Sanger sequencing confirmed proband’s homozygosity. For Patient 2, direct sequencing of the *ANTXR2* gene revealed a homozygous IVS11-1G>A (c.946-1G>A) mutation, leading to loss of a 3*′* splice site upstream exon 12, which likely impacts the downstream transmembrane domain causing reduced or complete loss of protein function (Figure 2A,B).

### 2.3. ANTXR2-deficient Organoids Are Not Affected in Growth and Polarization Potential

The ANTXR2-deficient patients described here, and multiple patients reported before [8,12,13], suffer from PLE. However, the pathogenesis of the intestinal phenotype has not been defined. Some HFS patients were reported to suffer from intestinal lymphangiectasia based on histological analysis [8,10,11,12,13], but it is unclear whether ANTXR2 deficiency leads to a primary defect of the epithelium as well. We generated intestinal organoids from Patient 1 in order to study the intrinsic properties of *ANTXR2*-mutant epithelial cells in an isolated system. The intestinal organoids were cultured in expansion medium (EM) to simulate the crypt-like progenitor cells, or in differentiation medium (DM) to differentiate the progenitors into mature enterocytes. 

**Figure 3:**
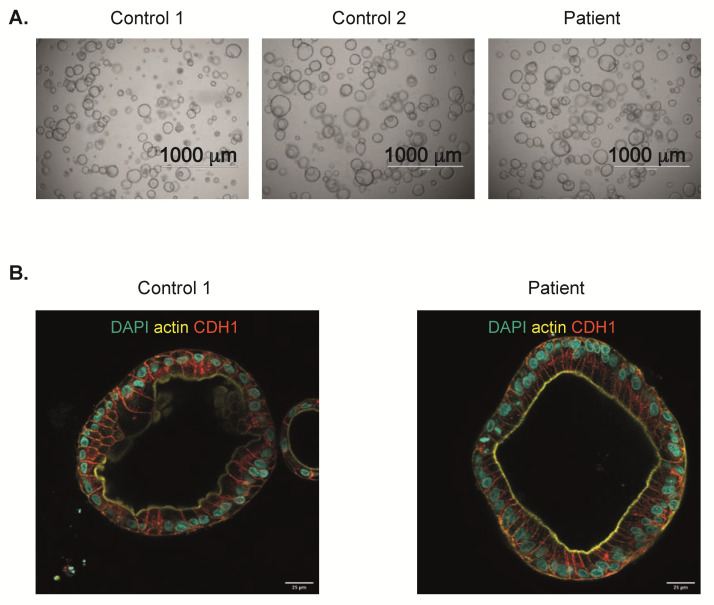
Normal growth and polarization of ANTXR2-deficient organoids. (**A**) Microscopic brightfield images of two controls and Patient 1 organoids grown for 7 days in EM. (**B**) Confocal staining for markers of enterocyte polarization. Actin (yellow) stains the apical membrane, E-cadherin (CDH1, red) stains the basolateral membrane and DAPI (blue) stains the nucleus.

The patient-derived organoids cultured in EM grew well, and could be passaged every week (Figure 3A), similarly to controls. As defective enterocyte polarization is a known cause of epithelial damage and malabsorption [15], we next studied if the *ANTXR2* c.155C > T mutation resulted in aberrant polarization of differentiated enterocytes. This was performed using phalloidin staining for actin filaments, a marker for the apical membrane in enterocytes [16], and E-cadherin staining as a marker for the basolateral membrane [17,18]. The resulting images show a discrete phalloidin staining on the apical side and proper localization of E-cadherin on the basolateral membrane for both control and patient organoids (Figure 3B), suggesting normal polarization of Patient 1 intestinal epithelium in vitro.

### 2.4. The c.155C>T Mutation Abolishes ANTXR2 Function

Previously, it has been shown that ANTXR2 is the receptor for *Bacillus anthracis* toxins [19]. Both the physiological and anthrax-toxin receptor function of ANTXR2 are dependent on ligand interaction with the MIDAS of the vWA domain in vitro [1], and HFS causing mutations have been shown to lose the ANTXR2 capacity to bind anthrax toxins [19]. These data indicate that the anthrax toxin assay provides a reliable assay to assess the physiological ANTXR2 function.

**Figure 4:**
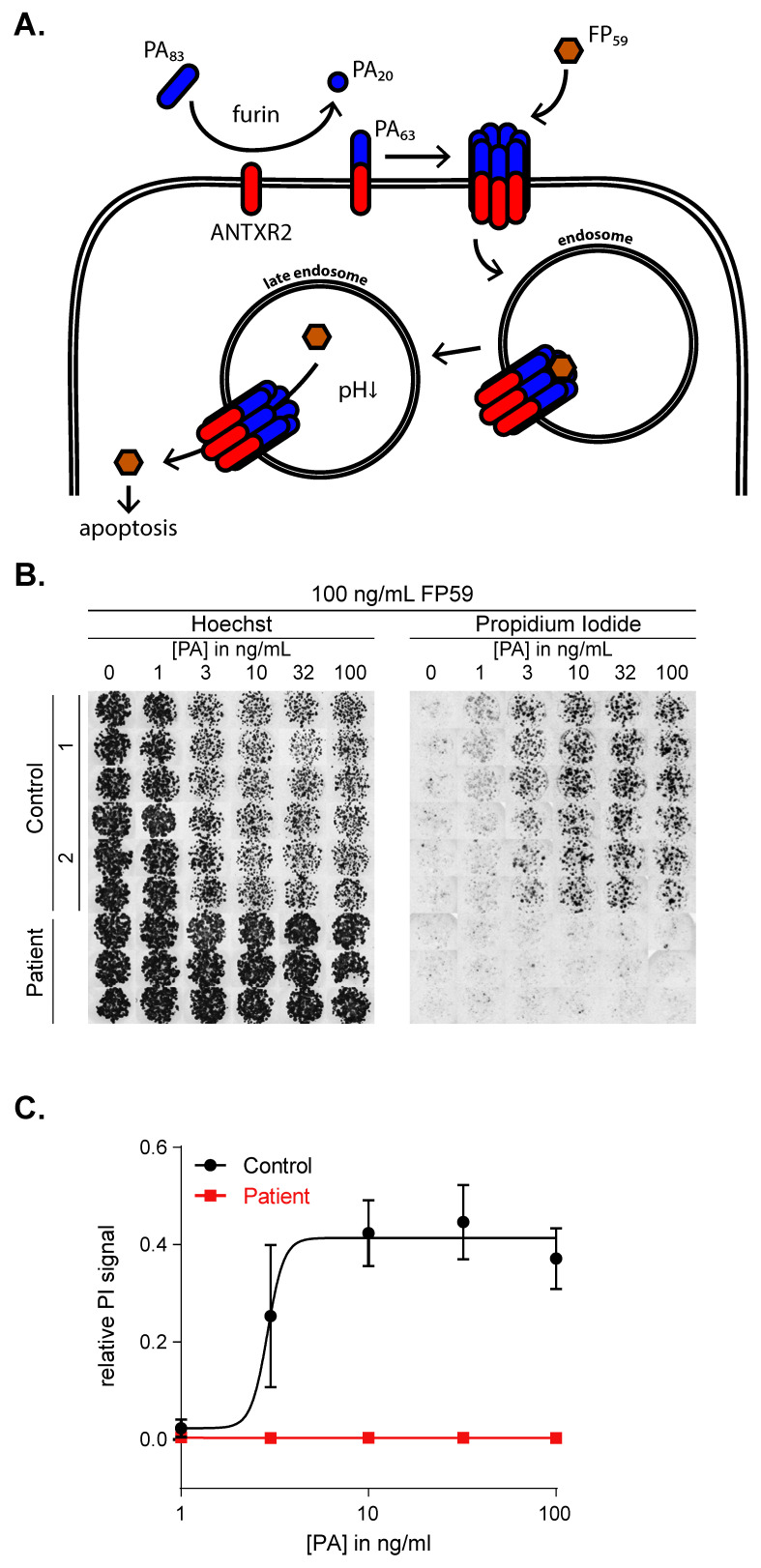
The c.155C>T *ANTXR2* mutation results in resistance to anthrax toxin-mediated cell death. (**A**) A schematic overview of the anthrax toxin mechanism of action, adapted from Deuquet et al. [20]. Anthrax toxin PA83 Is cleaved by furin to PA63, which binds to the vWA domain in ANTXR2. Oligomerization leads to pore formation in the cell membrane, which is promoted by the LF-derivative FP59. This allows internalization of the complex, where it is degraded in the late endosome by low pH. FP59 is then released in the cytosol and causes apoptosis. (**B**) Hoechst and PI stainings from the anthrax toxin assay performed on control and patient organoids grown on EM. (**C**) Quantification of the relative PI signal as a measure for cell death. Mean ± SD for *n* = 2 controls.

In the case of *Bacillus anthracis* infection, the anthrax toxin called protective antigen (PA) binds to the vWA domain in ANTXR2 or its isozyme anthrax toxin receptor 1 (ANTXR1) [8]. Ultimately, this leads to internalization of the toxin lethal factor (LF) and subsequently cell death (Figure 4A) [20,21]. Previous studies have exploited this mechanism to assess ANTXR2 function in vitro using recombinant PA and the highly toxic fusion protein FP59 in human cell lines [19]. It has been shown that the fusion protein FP59, which is a fusion between the PA-binding domain of anthrax LF and the catalytic domain of Pseudomonas aeruginosa exotoxin A, was at least 50-fold more toxic than LF in the presence of PA [22]. 

To confirm that the c.155C>T mutation impacts ANTXR2 protein function, we determined ANTXR2 ligand binding capacity by stimulating patient-derived and control organoids with an excess of FP59 and concentration range of PA. Since we did not detect any ANTXR1 expression in human organoids (Supplementary Figure S2), PA + FP59 toxicity in this system is completely dependent on intact ANTXR2 function. After stimulation with PA + FP59, we stained the organoids for propidium iodide (PI) (a membrane impermeant dye generally excluded from viable cells) and Hoechst (staining live cells brighter than dead cells). Cell death was determined by calculating the relative PI signal, which is the total area of PI fluorescence, normalized to each well’s total live and dead cell (PI + Hoechst) signal. These data show that healthy cells are highly sensitive to PA+FP59 induced cell death in a dose-dependent manner, while patient cells are completely resistant (Figure 4B,C). Collectively, our data indicate that the c.155C>T mutation induces loss of function of ANTXR2-dependent signaling, leading to complete resistance to *Bacillus anthracis* toxins.

### 2.5. ANTXR2 Deficiency Does Not Influence Organoid Monolayer Formation

As reported in earlier research, ANTXR2 was found to interact with its ligands laminin and collagen in endothelial cells [9] and fibroblasts [1,5]. ANTXR2 has therefore been suggested to play a role in cellular attachment to the basement membrane. To test if ANTXR2 deficiency impairs attachment of intestinal epithelium to the ECM as well, we cultured patient-derived, healthy, and CRISPR-Cas9 induced *ANTXR2^KO^* organoids as monolayers on transwell membranes, which were either coated with Matrigel, collagen IV, collagen VI or laminin. The CRISPR/Cas9 knock-out lines were generated using sgRNA oligomers targeted to the MIDAS domain (Supplementary Table S1) and selected for loss of function mutations with 10 ng/mL PA + 100 ng/mL FP59 (Supplementary Figure S3A,B). Successful CRISPR/Cas9 editing was confirmed using PCR and gel electrophoresis, which showed large insertions and deletions in many of the surviving organoid lines (Supplementary Figure S4). The monolayers were seeded in EM and differentiated using DM for seven days towards a mature enterocyte phenotype. Trans-epithelial electrical resistance (TEER) was measured in order to assess the integrity of cell monolayers and tight junction formation [23]. 

**Figure 5:**
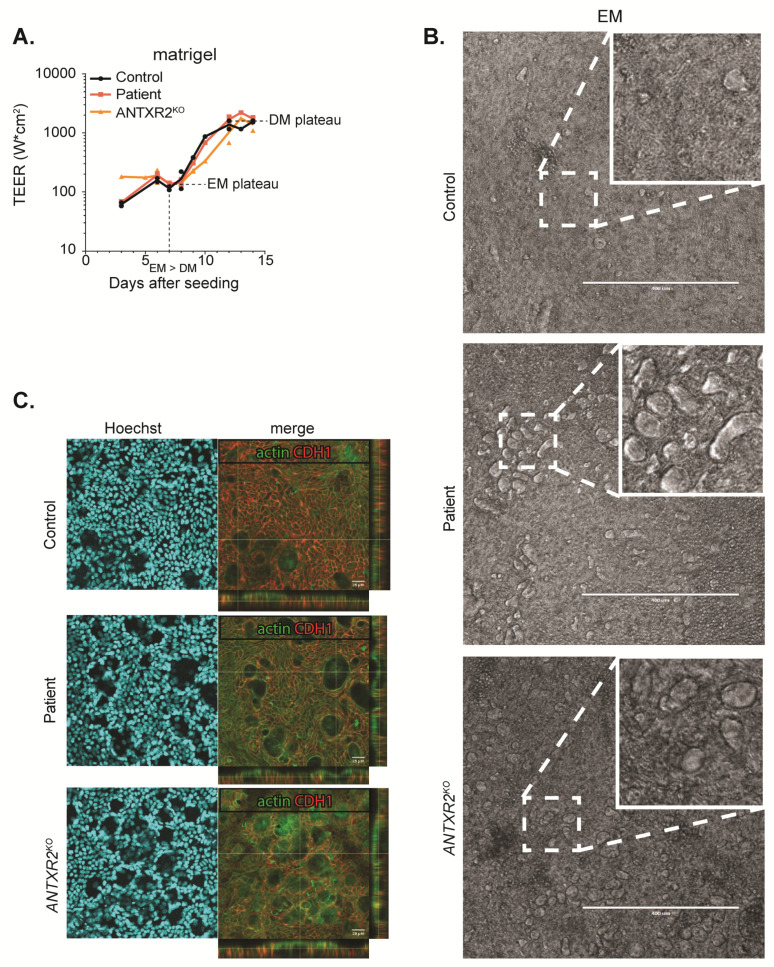
Normal monolayer cultures in ANTXR2-deficient organoids. (**A**) Trans-epithelial electrical resistance measured for control, patient, and *ANTXR2^KO^* organoids. (**B**,**C**) Monolayers were grown on EM for 7 days. (**B**) Microscopic brightfield images. Inserts show examples of blister formation and (**C**) confocal images for polarization markers, including actin (green, apical membrane), E-cadherin (CDH1, red, basolateral membrane) and DAPI (blue, nucleus).

Although ANTXR2-dependent signaling was completely lost in both Patient 1 and *ANTXR2^KO^* organoids, we did not observe a clear impairment of monolayer formation in the different organoid lines on either a matrigel coating (Figure 5 and Supplementary Figure S5) or any of the other coatings (data not shown). Microscopic observation of monolayer formation generally showed the formation of confluent monolayers (Supplementary Figure S5A), which was confirmed by comparable TEER of control, patient, and *ANTXR2^KO^* organoid lines (Figure 5A).

Although no clear impairment of monolayer formation was noted, we did observe small lesions, which were more prevalent in Patient 1 and *ANTXR2^KO^* lines after five days of culture in EM (Figure 5B,C). We studied these lesions in more detail using confocal microscopy, and found that these lesions are blister-like fluid filled structures covered with actin positive cell surface. Since E-Cadherin (CDH1) basolateral staining was present all around these blisters, they seem to be fully surrounded by the epithelial cells. Soon after initiating differentiation of the monolayers, these structures disappeared, resulting in smooth, polarized epithelium in control, patient, and *ANTXR2^KO^* organoids alike (Supplementary Figure S5A,B). Collectively, our studies suggest that the disease phenotype of ANTXR2-deficient patients is not caused by intrinsic defects of the intestinal epithelium.

### 2.6. Abnormal Duodenal Collagen Deposition VI in ANTXR2 Deficiency

**Figure 6:**
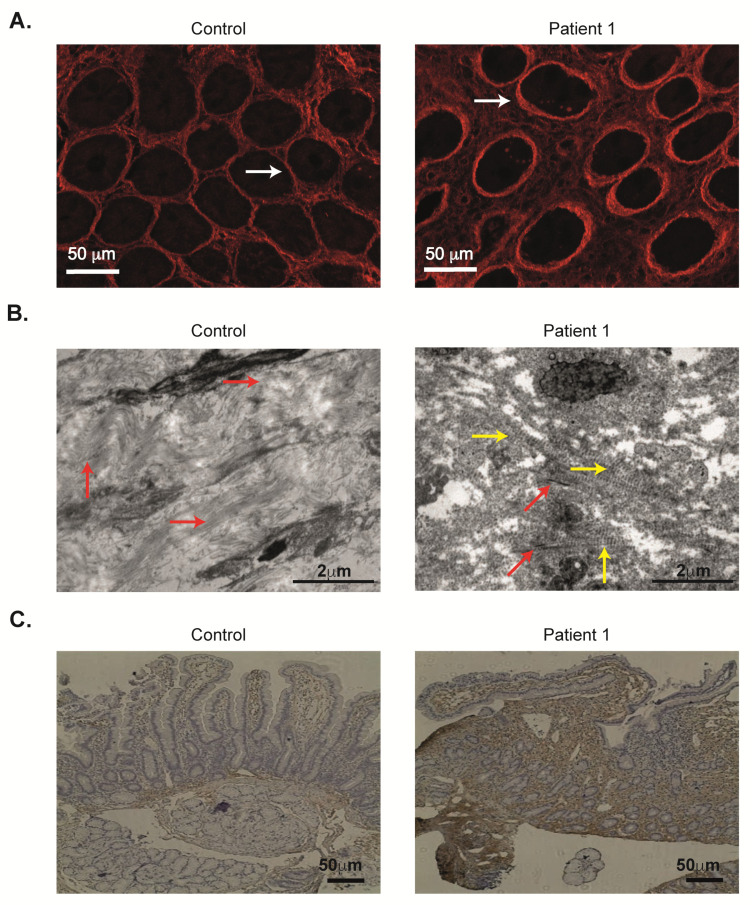
Abnormal extracellular matrix morphology in duodenum of ANTXR2-deficient patients. (**A**) Second harmonic generation imaging of duodenal FFPE samples. Red signifies collagen. White arrows point to crypt wall. (**B**) Duodenal electron microscopy images (×15,000) from control subject (infant evaluated for chronic diarrhea) and Patient 1. Red arrows point to collagen I and yellow arrows point to collagen VI. (**C**) Collagen VI staining (brown colored) in duodenal sections.

Burgi and colleagues have recently reported that loss of ANTXR2 leads to collagen VI accumulation in the skin and generation of nodules [1]. They identified ANTXR2 as a receptor that binds collagen VI and mediates its intracellular degradation [1]. We initially performed second harmonic generation (SHG) imaging, which visualizes non-labelled fibrillar ECM proteins, such as collagen [24,25]. SHG studies demonstrated widening of the spaces between the crypts by collagen deposition (Figure 6A). In order to quantify these changes, distance between the center of the crypts to adjacent crypts, and number of crypts per slide were calculated. As can be seen in Figure S6, the distance between the crypts was longer and fewer crypts per slide were demonstrated, suggesting widening of the interstitial space between the crypts. Moreover, the duodenal crypt walls were lined with a thicker layer of collagen compared with controls, as pointed out by arrows in Figure 6A. Next, electron microscopy examination of duodenal biopsies from Patient 1 revealed the presence of long spacing collagen inside the connective tissue, typical of collagen VI (Figure 6B). These are fibrils characterized by a banding periodicity of approximatively 120–200 nm. In a few areas bundles of collagen I were observed, but to a much lesser extent compared with control (Figure 6B). Moreover, the architecture of the ECM was less dense in the patient’s sample. Finally, collagen VI expression in duodenal samples from these two ANTXR2-deficient patients was markedly elevated, compared with controls (Figure 6C, Supplementary Figure S6). Overall, these studies indicate abnormal ECM structure in the duodenum, which may lead to PLE and associated clinical phenotype.

## 3. Discussion

With the expanding use of advanced genetic sequencing studies for patients with unique clinical phenotypes, several monogenic disorders causing intestinal lymphangiectasia and PLE have been identified in recent years, resulting from mutations in diacylglycerol acyltransferases 1 (*DGAT1*) [26,27], Plasmalemma Vesicle Associated Protein (*PLVAP*) [28,29], *CD55* [30,31], collagen and calcium binding EGF domains 1 (*CCBE1*) [32], among others. These patients present with severe diarrhea and PLE, usually in the first months of life that is often fatal, although recently treatment with eculizumab was shown to be highly effective in reversing the gastrointestinal phenotype in patients with CD55 deficiency [30,33]. Moreover, some patients with DGAT1 mutations improve over time and are able to wean off TPN or remain stable with a low fat or fat-free diet [27].

HFS resulting from *ANTXR2* mutations is another multi-systemic disease that manifests with chronic diarrhea and failure to thrive in the severe forms [5,8]. In some of the published reports HFS patients were noted to exhibit signs of PLE and intestinal lymphangiectasia [8,10,11,12,13]. Similar to previous reports, the current patients also exhibited severe diarrhea that was unresponsive to various nutritional interventions and did not improve over time. Both patients also presented with typical clinical features that should hint for this diagnosis, including unique hyperpigmented macules over bony prominences (specifically, the metacarpophalangeal joints and medial malleoli) [34], thickened skin and gingival hypertrophy.

Unfortunately, treatment of HFS patients is currently limited to supportive care [6,8]. For patients with mild phenotypes this includes pain management and physiotherapy for joint stiffness, and surgical resection of skin and oral lesions in case of extreme discomfort or ulceration. For patients suffering from more severe manifestations with involvement of the intestine and other internal organs, there is currently no treatment and palliative care is suggested [8]. A previous study on HFS-patient derived fibroblasts showed that pharmacological inhibition of the proteasome partially rescued the functional protein from ER-associated degradation in three out of five selected patient cells [35]. Interestingly, the level of rescue correlated with disease severity and was weaker in cells from patients with severe phenotypes [35]. Therefore, proteasomal inhibitors such as bortezomib may be beneficial in selected patients with *ANTXR2* mutations affecting protein structure.

Mechanistically, PLE can be the result of alterations in intestinal lymph fluid flow, mucosal erosions or inherent epithelial defects [36]. The identification of monogenic causes of PLE has underlined different pathways that are involved in protein homeostasis in the gut. Using patient-derived organoids, we have previously shown that DGAT1 deficiency results in aberrant lipid metabolism and increased susceptibility to lipid-induced toxicity, which may explain the PLE phenotype [27]. Alternatively, mutated *PLVAP* leads to aberrant formation of the diaphragms of endothelial fenestrae, resulting in vascular leakage [28]. In CD55 deficiency, a possible link between the complement system and intestinal mucosal homeostasis has been identified [37]. CD55 is an important regulator of the complement cascade; patients with deleterious *CD55* mutations exhibited enhanced deposition of C5b-9, the membrane attack complex, in sub-mucosal arterioles in the duodenum, which may explain the enhanced intestinal protein loss [30,31]. Finally, patients with Hennekam syndrome, resulting from mutations in *CCBE1* [32], *FAT4* [38] or *ADAMTS3* [39], exhibit diffuse lymphatic dysplasia which also affects lymph flow in the gut.

Here we show that PLE in patients with HFS does not result from intrinsic epithelial defects. Although the ANTRX2-deficient organoids failed to respond to the Anthrax toxin, polarization, proliferation, differentiation and monolayer formation of patient-derived or *ANTXR2^KO^* organoids were comparable to control organoids. The electron microscopy and SHG studies demonstrate marked changes in ECM structure in the gut, and are in line with increased collagen VI expression. These changes in the interstitial matrix, underlying the epithelium and basement membrane, most likely are associated with functional biochemical changes in the tissue. Taken together with the organoid analysis, these results suggest that the interstitial matrix may be the dominant tissue component leading to barrier dysfunction in these patients, rather than the epithelium. Nevertheless, the functional effects of different mutations affecting processing of ANTXR2 and/or localization should be determined, in order to define whether they have an effect on ECM structure and epithelial cell function.

Recently, Burgi and colleagues reported that collagen VI accumulates in skin nodules of patients with HFS and in the uterine of *Antxr2^−/−^* mice [1]. They also identified that ANTXR2 can bind collagen VI and targets it for intracellular degradation [1]. These observations, as well as our studies in the gut, associate ECM disorders with development of PLE. It would be interesting to determine whether patients with milder forms of HFS (grades 1 and 2) also exhibit ECM changes and PLE, but to a lesser extent, than those identified in this study.

The organoid studies using patient-derived biopsies were limited to a single case, given the rarity of this disorder and the difficulty of obtaining research biospecimens from very young and sick patients. Nevertheless, we have shown that genetic modification using CRISPR/Cas9 is a feasible approach to model ANTXR2 deficiency in vitro. We successfully generated random indel knock-outs using combinations of sgRNAs, and selected them based on their resistance to anthrax-toxin cytotoxicity. This functional selection was both very sensitive and specific, since anthrax toxins are strictly receptor specific and are cytotoxic at very low concentrations. The low efficiency of homology-directed repair-guided gene editing can therefore likely be overcome and would be a feasible approach to model patient-specific mutations in culture. Such studies can allow drug screens including high-throughput approaches to specifically restore ANTXR2 function.

In conclusion, we demonstrate that the PLE phenotype in patients with HFS grade 4 does not result from intrinsic epithelial defects. Rather, the PLE phenotype in patients with HFS grade 4 is associated with abnormal ECM composition. Loss of ANTXR2-dependent signaling leads to a multi-systemic deposition of collagen that has severe clinical manifestations which can be lethal. Additional studies are required to understand whether approaches that specifically guide collagen VI for lysosomal degradation can alleviate the clinical features and ECM alterations in this rare disease.

## 4. Materials and Methods

### 4.1. Study Approval

The study was approved by the responsible local ethics committees at Sheba Medical Center (SMC-3312-16) and the University Medical Center Utrecht (METC Utrecht, 10-402, 15 August 2010). Parents of participating subjects provided written informed consent for the collection of samples and subsequent analysis. All co-authors had access to the study data and had reviewed and approved the final manuscript. All methods were carried out in accordance with relevant guidelines and regulations.

### 4.2. DNA Sequencing

Whole exome sequencing was performed on Patient 1 and results were validated by targeted Sanger sequencing. Patient 2 underwent targeted Sanger sequencing.

### 4.3. Organoid Culture

Organoids were generated from duodenal biopsies obtained from two healthy controls and Patient 1 during EGD for diagnostic purposes. Control 1 was a four-year-old male at the time the biopsy was taken and control 2 a four-year-old female. Organoid cultures were established from crypts which were isolated from the biopsies as reported before [27]. In brief, crypts were isolated from intestinal biopsies by dissociation with 10 mM ethylenediaminetetraacetic acid in phosphate buffered saline (PBS) with dithiothreitol for one hour and collected by pipetting vigorously. Isolated crypts were resuspended in medium without growth factors (GF-), consisting of Advanced DMEM/F12, 100 U/mL penicillin-streptomycin, 10 mM HEPES and Glutamax. Matrigel (Corning, Tewksbury, MA, USA; Matrigel Matrix Growth Factor Reduced, Phenol Red-Free) was added at a final concentration of 70% and plated on pre-warmed cell culture 24- or 96-well plates. After Matrigel polymerization, organoid EM was added consisting of GF-medium, 50% WNT-3A-conditioned medium, 20% R-Spondin-1-conditioned medium, 10% Noggin-conditioned medium, 50 ng/mL murine EGF, 10 mM nicotinamide, 1.25 mM N-acetyl, B27, 500 nM TGF-β inhibitor A83-01 and 10 µM P38 inhibitor SB202190. Organoids were cultured at 37 °C and 5% CO_2_ and medium was refreshed every 2–3 days. The organoids were passaged as single cells (TrypLE Express, Thermo Scientific, Waltham, MA, USA), counted and seeded as 250 cells/μL in the Matrigel mix. After passaging, 10 μM ROCK inhibitor Y-27632 was added for the first 2–3 days of the culture. To induce differentiation, organoids were cultured in DM for five days, which consists of EM lacking WNT-3A-conditioned medium, nicotinamide and SB202190 [27].

Polarized organoid monolayers were grown as described before [16] with some alterations. After 7 days of 3D culture in EM, organoids were harvested and processed into single cells by resuspension and subsequently incubation with TrypLE Express for 5–10 min. Polyester Transwell membranes (6.5-mm Transwell with 0.4-μm pore; Corning, Tewksbury, MA, USA) were coated for at least 1 h at room temperature (RT) with Matrigel diluted 1:40 in PBS with calcium chloride and magnesium chloride, 10 μg/mL collagen IV (Sigma-Aldrich, St. Louis, MO, USA), 5 μg/mL collagen VI (Sigma-Aldrich) or 2 μg/mL laminin (Sigma-Aldrich). After removal of coating, 2.5 × 10^5^ to 4.0 × 10^5^ cells were seeded in EM supplemented with 10 μM ROCK inhibitor (ROCKi, Y-27632, Abcam, Cambridge, UK). Trans-epithelial electrical resistance was determined daily by using the EVOM2 (World Precision Instruments, Sarasota, FL, USA). After 3 days, the medium was replaced by EM without ROCKi. When cells were confluent and the TEER value reached a plateau phase, differentiation was induced by replacing the EM with DM. When the TEER value reached the next plateau phase, usually after 7 days of culturing in DM, the cells were considered fully differentiated.

### 4.4. Quantitative Real-Time Polymerase Chain Reaction

RNA was isolated using TRIzol LS Reagent from organoids grown in either EM for 10 days or EM for 5 days and subsequently DM for five days according to the manufacturer’s protocol. cDNA was synthesized using the iScript cDNA synthesis kit and amplified using SYBR green supermix in a Light Cycler96 (Bio-Rad, Hercules, CA, USA) using a 2-step program, with an annealing temperature of 62 °C for 40 cycles and subsequent melt curve measurements from 95 to 55 °C, according to the manufacturer’s protocol. The comparative Ct method was used to quantify the data. The relative quantity was defined as 2^−ddCt^. Primers are listed in Supplementary Table S2; *HP1BP3* was used as housekeeper gene.

### 4.5. Confocal Microscopy

Differentiated organoids and organoid-derived monolayers were fixed in 4% buffered formaldehyde for 30 min at RT, and permeabilized using 0.2% Triton-X100 for 30 min at 4 °C, as previously described [27]. After washing with PBS, cells were blocked for 1 h at RT with 5% BSA in phosphate buffered saline with Tween, followed by incubation with phalloidin-TRITC (Sigma-Aldrich) diluted 1:100 and Alexa Fluor 647 mouse anti-E-cadherin (BD Biosciences, San Jose, CA, USA) diluted 1:100 for 1 h at RT in the dark. Cells were washed and stored in PBS after counterstaining with DAPI (Sigma-Aldrich) in PBS for 5 min. Imaging of the organoids and monolayers was performed using a Leica SP8X laser-scanning confocal microscope outfitted with a white light laser [27].

### 4.6. Anthrax Toxin Cytotoxicity Assay

Organoids were cultured in Matrigel in EM for 5 days in black clear-bottom 96-well imaging plates (Corning), followed by treatment with serial dilutions of PA ranging 0–100 ng/mL (Kerafast Inc., Boston, MA, USA) and 100 ng/mL FP59 (Kerafast Inc.) in EM for 2 days. The treated organoids were washed with PBS and stained with Hoechst 5 µg/mL (Sigma-Aldrich) and PI 0.1 mg/mL (Thermo Fisher) in PBS at RT for 15 min, and imaged using an inverted Olympus IX53 (Olympus, Tokyo, Japan) epifluorescence microscope. Resulting images were analyzed using Fiji/ImageJ for total area of fluorescence of PI [40,41]. To calculate the relative PI signal, the total area of PI fluorescence was normalized to each well’s total live and dead cell signal according to the formula *R_x_* = *P_x_*/(*P_x_* + *H_x_*) where P is the total area of PI fluorescence and H the total area of Hoechst fluorescence for PA concentration ‘*x*’, where *x* ranges [0, …, *n*]. The resulting relative PI signal was averaged across technical triplicates for each line of intestinal organoids, and three biological replicates were included in the control group. The relative PI signal for the three biological replicates were plotted using Graphpad Prism (Graphpad Software, San Diego, CA, USA) and fitted with a non-linear regression with variable slope.

### 4.7. CRISPR-Cas9 Knockout of ANTXR2

*ANTXR2* sgRNAs were designed using the design tool at “http://mit.crispr.edu” within 100 base pairs from the patient’s mutation (Supplementary Table S1). The resulting sgRNAs were cloned into the pSpCas9(BB)-2A-Puro (PX459, Addgene #62988) vector as described before [42]. Prior to transfection, the organoids were primed by removing WNT3A- and R-Spondin-1-conditioned media and addition of 5 μM CHIR99021, 1.25% DMSO and 10 μM Y-27632 to the culture medium as described previously [43]. Two different sgRNA plasmid mixes (sgRNA#1+2 and sgRNA#3+4) overlapping the patient’s mutated region were used for transfection, intended to obstruct the MIDAS motif of the vWA domain. The transfection was performed on 1 × 10^6^ single cells in 80 μL Opti-MEM (Gibco, Thermo Fisher, Waltham, MA, USA) mixed with 10 μL *ANTXR2* sgRNA construct plasmid (1 μg/μL). Transfection was performed in a NEPA21 Electroporator (Nepa Gene, Chiba, Japan) with settings as described previously [43]. After transfection, priming medium was used for 5 days before replacement by standard EM. Since the patient-derived organoids were completely resistant to cytotoxicity induced by anthrax toxin PA and FP59, transfected cells were selected by treatment with 10 ng/mL PA and 100 ng/mL FP59. Of the surviving organoid lines, a ca. 500 bp region surrounding ANTXR2 exon 2 was amplified using PCR primers (forward: TGTGGTTTGCATTTCCTGCG, reverse: TGCAATACGACCTTGAGGCA) and checked for insertions/deletions using agarose gel electrophoresis.

### 4.8. Second Harmonic Generation Imaging

10 µm-thick formalin-fixed paraffin-embedded (FFPE) sections of duodenum and skin from ANTXR2-deficient patients and two controls were deparaffinised and unstained collagen was imaged using SHG under a two-photon microscope. Deparaffinisation was performed by dewaxing of slides using W-Cap solution (Leica Biosystems GmbH, Wetzlar, Germany) in 75 °C for 20 min, then washed twice in ddH_2_O, and remained to cool down to RT. Following, the slides were dried and mounting solution (Immu-MountTM, Thermo Fisher, Waltham, MA, USA) was applied. Finally, the slides were covered with a coverslip and were allowed to dry overnight. The slides were imaged under a Leica TCS SP8 multiphoton (MP) system using the MP laser set to a 855 nm wavelength. The reflected SHG signal of fibrillary collagen was detected at 427.5 nm.

### 4.9. Electron Microscopy

Duodenal biopsies were initially fixed in 2.5% glutaraldehyde in 0.1M buffered cacodylate for 24 h, then with in 1% osmium tetroxide for 1 h, following dehydration in a series of increasing ethanol concentrations, and finally embedded in epoxy resin—Agar mix (Agar Scientific LTD, Essex, UK) for 2 days at 60 °C. Next, semi-thin sections were prepared from the block and relevant areas were selected for ultra-thin sections. These sections were then stained with uranyl acetate and lead citrate. Examination was performed in a Jeol—1200 EX transmission electron microscope (Jeol, Peabody, MA, USA).

### 4.10. Immunohistochemistry

Tissues were fixed in 4% paraformaldehyde and embedded in paraffin, and serial 5-μm sections were prepared from the whole biopsy for immunohistochemistry. Sections were incubated for 1 h with a primary antibody to Collagen VI (Rabbit Polyclonal, ab6588, Abcam, Dilution 1:100) at RT in a humidity chamber, washed and incubated with fluorescence secondary antibody for 1 h [(Cy3-AffiniPure Donkey Anti-Rabbit IgG (H + L) (711-165-152, 1:200); Jackson Immunoresearch Laboratories] and DAPI as a counterstain. Tissue exposed to secondary antibody only was used as negative control.

## Supplementary Materials

The following are available online at https://www.mdpi.com/1422-0067/21/21/8200/s1. Figure S1. Identification of intestinal lymphangectasia in the index patient. Figure depicts D2-40 stain for lymphatic vessels in duodenal sections from a control subject and Patient 1. Upper panel, low maginifcation; lower panel, high maginifcation. Figure S2. RT-qPCR results from control and patient-derived organoids. Figure S3. Organoid sensitivity to anthrax toxins. Images from (A) control, (B) patient-derived, and seven independent clonal ANTXR2KO organoids grown in EM with 10 ng/mL PA + 100 ng/mL FP59 for two days. Figure S4. Conformation of indel mutations in ANTXR2KO organoid lines. Figure displays gel electrophoresis of ANTXR2 exon 2, confirming indel mutations in many of the shown surviving organoid KO lines. Figure S5. Microscopic images from organoid monolayers cultures. (A) Brightfield images from organoid monolayers in EM and DM. (B) Confocal images of enterocyte polarization markers on monolayers on DM, day 12. Actin (green, apical membrane), E-cadherin (CDH1, red, basolateral membrane) and DAPI (blue, nucleus). Figure S6. Abnormal ECM composition in ANTXR2 deficiency. Figure depicts (A) distances between center of 2 adjacent crypts, (B) mean number of crypts per slides and (C) percent of positive COL6A stain. For all studies data from 3 different sections was obtained, and the mean value is presented in the figures. Table S1. sgRNA oligomers cloned into PX459. Table S2. Primer sets for RT-qPCR.

## Abbreviations

ANTXR2	anthrax toxin receptor 2
CMG2	capillary morphogenesis protein 2
DM	differentiation media
ECM	extracellular matrix
EGD	esophagogastroduodenoscopy
EF	edema factor
EM	expansion media
GF	growth factors
FFPE	formalin-fixed paraffin-embedded
H&E	hematoxylin and eosin
HFS	hyaline fibromatosis syndrome
ISH	infantile systemic hyalinosis
LF	lethal factor
MIDAS	metal ion-dependent adhesion site
MP	multiphoton
PA	protective antigen
PBS	phosphate buffered saline
PI	propidium iodide
PLE	protein losing enteropathy
ROCKi	ROCK inhibitor
RT	room temperature
SHG	second-harmonic generation
TEER	trans-epithelial electrical resistance
TPN	total parenteral nutrition
vWA	von Willebrand A
