# Supplementary material for: Enhanced Collagen Deposition in the Duodenum of Patients with Hyaline Fibromatosis Syndrome and Protein Losing Enteropathy
Source: Int J Mol Sci. 2020 Nov 2;21(21):8200. doi: 10.3390/ijms21218200 (PMC7662532; doi:10.3390/ijms21218200)
Supplement: Supplementary file 1 [file ijms-21-08200-s001.pdf]

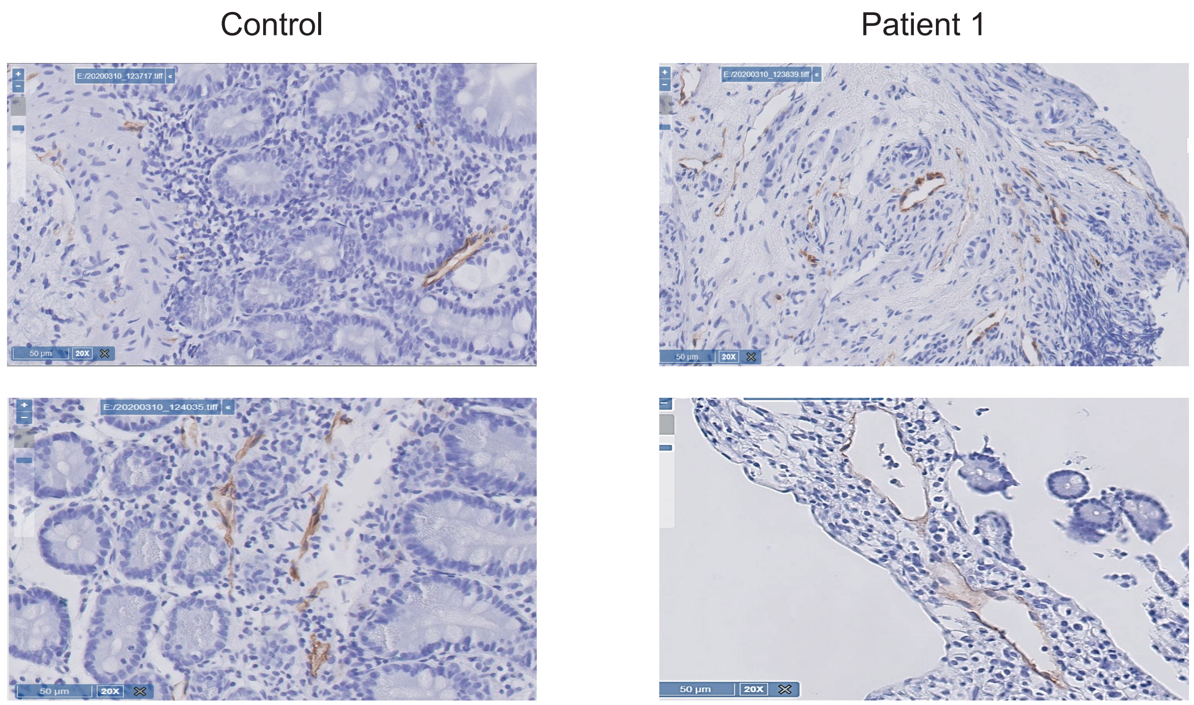

Supplementary Figure S1. Identification of intestinal lymphangectasia in the index patient. Figure depicts D2-40 stain for lymphatic vessels in duodenal sections from a control subject and Patient 1. Upper panel, low maginifcation; lower panel, high maginifcation.

A.

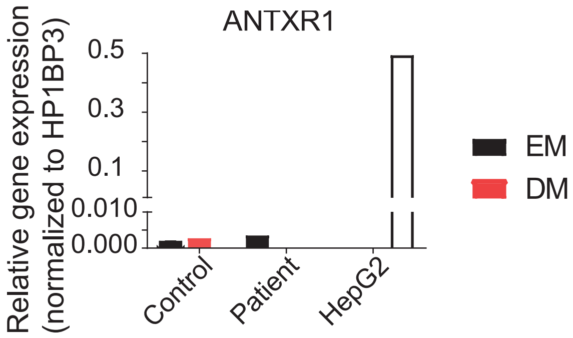

B.

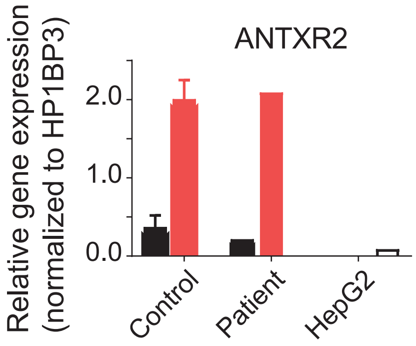

Supplementary Figure S2. RT-qPCR results from control and patient-derived organoids.

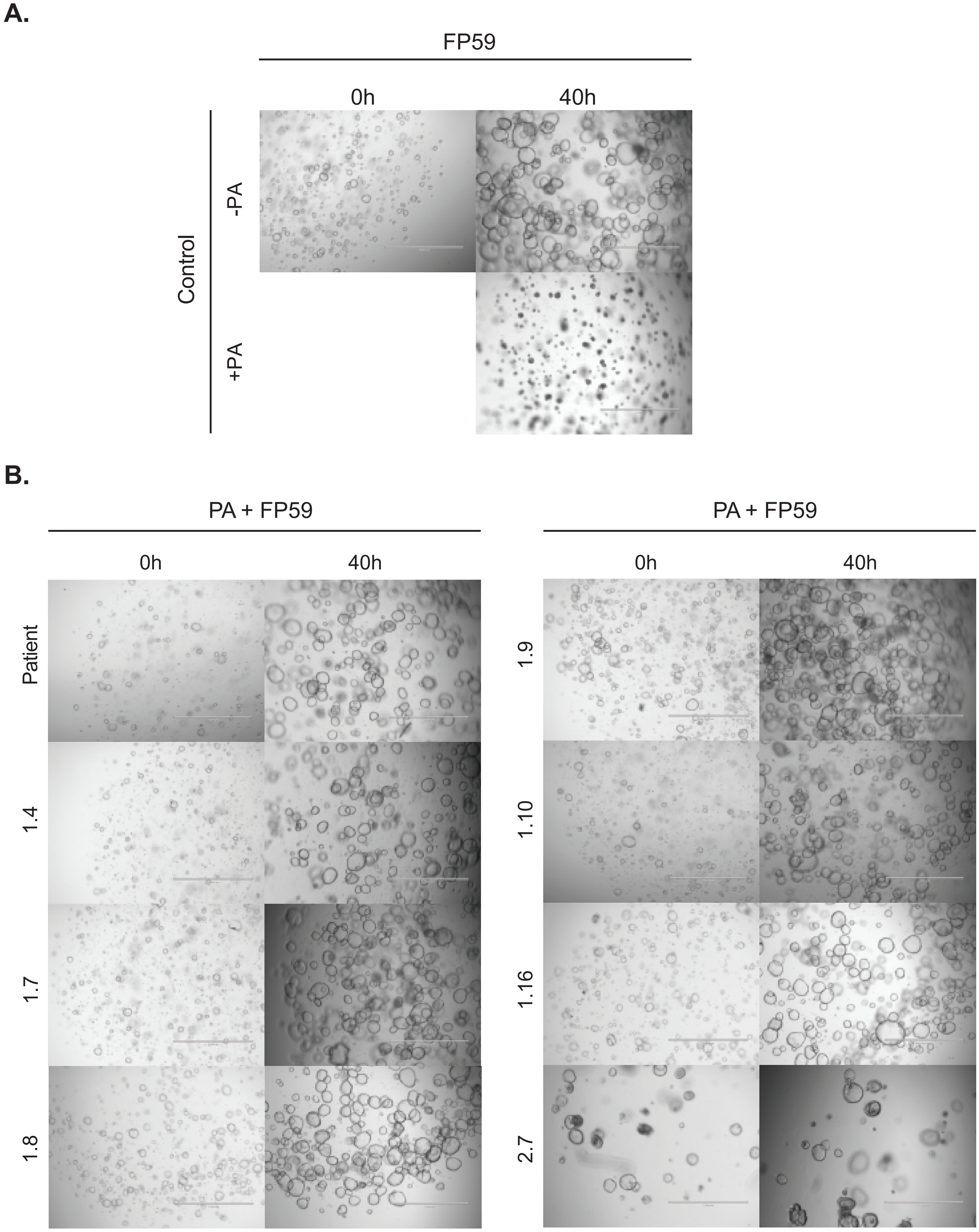

Supplementary Figure S3. Organoid sensitivity to anthrax toxins. Images from (**A**) control, (**B**) patient-derived, and seven independent clonal ANTXR2^{KO} organoids grown in EM with 10 ng/ml PA + 100 ng/ml FP59 for two days.

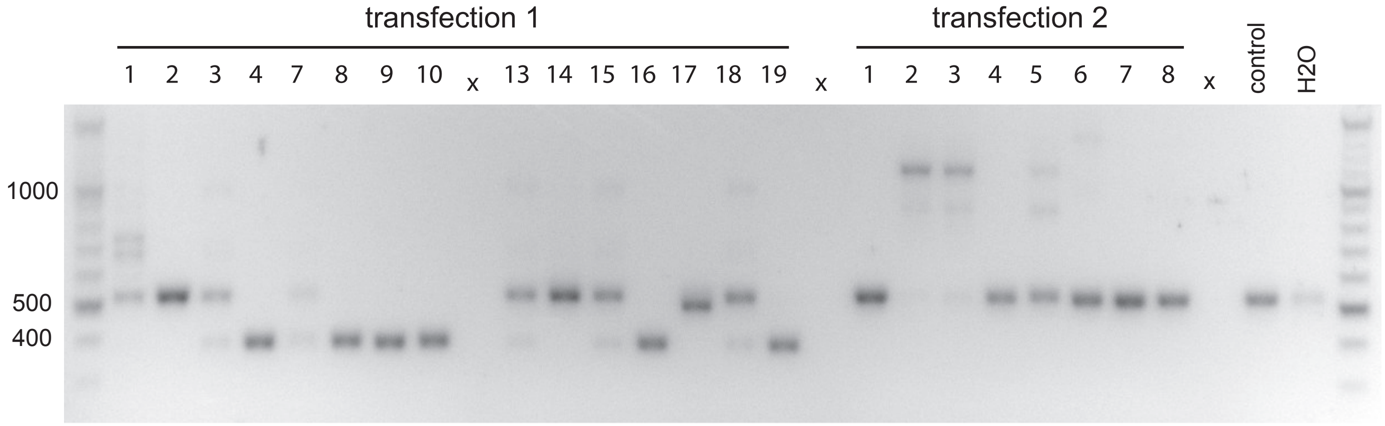

Supplementary Figure S4. Conformation of indel mutations in ANTXR2^{KO} organoid lines. Figure displays gel electrophoresis of ANTXR2 exon 2, confirming indel mutations in many of the shown surviving organoid KO lines.

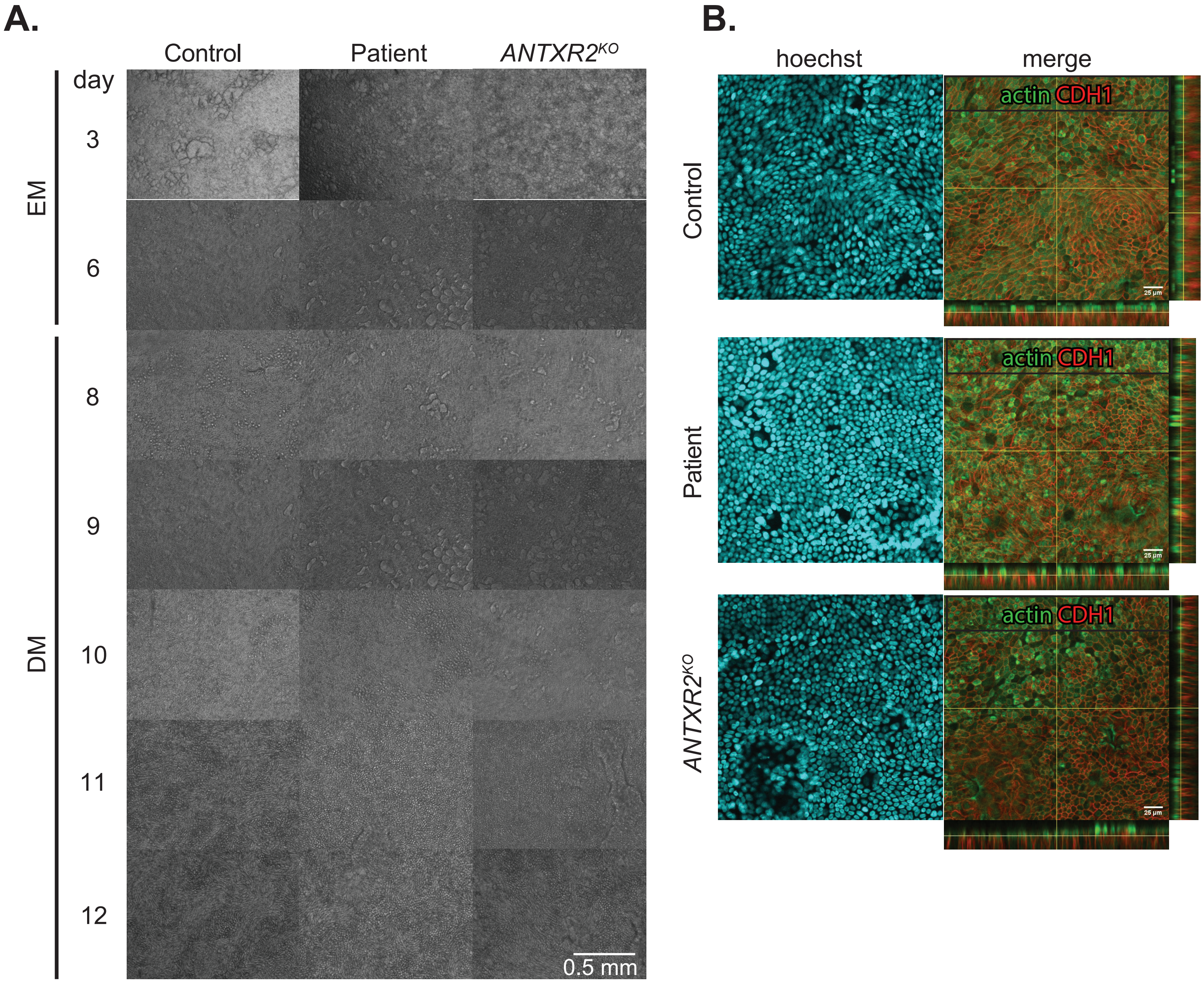

Supplementary Figure S5. Microscopic images from organoid monolayers cultures. (**A**) Brightfield images from organoid monolayers in EM and DM. (**B**) Confocal images of enterocyte polarization markers on monolayers on DM, day 12. Actin (green, apical membrane), E-cadherin (CDH1, red, basolateral membrane) and DAPI (blue, nucleus).

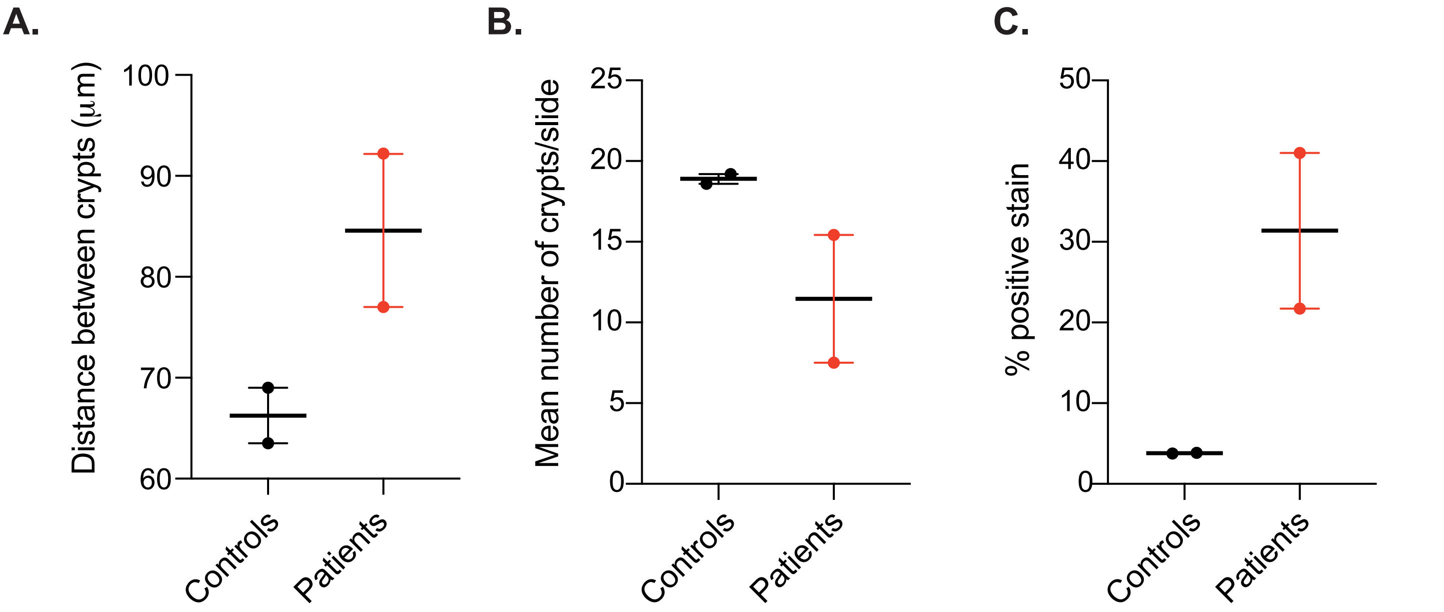

Supplementary Figure S6. Abnormal ECM composition in ANTXR2 deficiency. Figure depicts (**A**) distances between center of 2 adjacent crypts, (**B**) mean number of crypts per slides and (**C**) percent of positive COL6A stain. For all studies data from 3 different sections was obtained, and the mean value is presented in the figures.

Supplementary Tables

Supplementary Table S1. sgRNA oligomers cloned into PX459.

	sgRNA name	PAM-strand (5'->3')	Complementary strand (5'->3')
Set 1	sgRNA#1	caccgTTCTTCAATGCCACGAACAT	aaacATGTTCGTGGCATTGAAGAAc
	sgRNA#2	caccgTAATTTCGTACAGCAACTTG	aaacCAAGTTGCTGTACGAAATTAc
Set 2	sgRNA#3	caccgTCAATTTCATCTTTCAGGTC	aaacGACCTGAAAGATGAAATTGAc
	sgRNA#4	caccgATCTTTCAGGTCTGGGAGTG	aaacCACTCCCAGACCTGAAAGATc

Nucleotide bases in uppercase indicate the 20-bp gene specific seed region of the final sgRNA molecule. Bases in lowercase indicate the BbsI restriction sites used to clone the double stranded oligos into the PX459 vector.

Supplementary Table S2. Primer sets for RT-qPCR.

Target	Forward primer (5'->3')	Reverse primer (5'->3')
HP1BP3	CCCACGTCCCAAGATGGAT	CTGATGCACCACTCTTCTGGAA
ANTXR1 (CMG1)	CGGATTGCGGACAGTAAGGAT	TCCTCTCACGACAACTTGAAATG
ANTXR2 (CMG2)	GCTTTGTCCTGCACCTATCCT	CCACATCAAACCGATCCCCA
